# Evaluation of a Web-Based Tailored Nursing Intervention (TAVIE en m@rche) Aimed at Increasing Walking After an Acute Coronary Syndrome: A Multicenter Randomized Controlled Trial Protocol

**DOI:** 10.2196/resprot.6430

**Published:** 2017-04-27

**Authors:** John William Kayser, Sylvie Cossette, José Côté, Anne Bourbonnais, Margaret Purden, Martin Juneau, Jean-Francois Tanguay, Marie-Josée Simard, Jocelyn Dupuis, Jean G Diodati, Jean-Francois Tremblay, Marc-André Maheu-Cadotte, Daniel Cournoyer

**Affiliations:** ^1^ Université de Montréal, Faculty of Nursing Montréal, QC Canada; ^2^ Montreal Heart Institute Research Center and Université de Montréal Montréal, QC Canada; ^3^ Research Center of the Centre Hospitalier de l’Université de Montréal Montréal, QC Canada; ^4^ Research Center of the Institut universitaire de gériatrie de Montréal Montréal, QC Canada; ^5^ McGill University, Ingram School of Nursing Montréal, QC Canada; ^6^ Jewish General Hospital Centre for Nursing Research Montréal, QC Canada; ^7^ Integrated Health and Social Services Centres, l'Est de l'Île de Montréal Montréal, QC Canada; ^8^ Hôpital du Sacré-Cœur de Montréal Montréal, QC Canada; ^9^ Hôpital Maisonneuve-Rosemont Montréal, QC Canada; ^10^ Montreal Health Innovations Coordinating Center Montréal, QC Canada

**Keywords:** acute coronary syndrome, secondary prevention, physical activity, walking, Internet, computer-tailored, eHealth, Strengths-Based Nursing Care, Self-Determination Theory, nursing intervention

## Abstract

**Background:**

Despite the health benefits of increasing physical activity in the secondary prevention of acute coronary syndrome (ACS), up to 60% of ACS patients are insufficiently active. Evidence supporting the effect of Web-based interventions on increasing physical activity outcomes in ACS patients is growing. However, randomized controlled trials (RCTs) using Web-based technologies that measured objective physical activity outcomes are sparse.

**Objective:**

Our aim is to evaluate in insufficiently active ACS patients, the effect of a fully automated, Web-based tailored nursing intervention (TAVIE en m@rche) on increasing steps per day.

**Methods:**

A parallel two-group multicenter RCT (target N=148) is being conducted in four major teaching hospitals in Montréal, Canada. An experimental group receiving the 4-week TAVIE en m@rche intervention plus a brief “booster” at 8 weeks, is compared with the control group receiving hyperlinks to publicly available websites. TAVIE en m@rche is based on the Strengths-Based Nursing Care orientation to nursing practice and the Self-Determination Theory of human motivation. The intervention is centered on videos of a nurse who delivers the content tailored to baseline levels of self-reported autonomous motivation, perceived competence, and walking behavior. Participants are recruited in hospital and are eligible if they report access to a computer and report less than recommended physical activity levels 6 months before hospitalization. Most outcome data are collected online at baseline, and 5 and 12 weeks postrandomization. The primary outcome is change in accelerometer-measured steps per day between randomization and 12 weeks. The secondary outcomes include change in steps per day between randomization and 5 weeks, and change in self-reported energy expenditure for walking and moderate to vigorous physical activity between randomization, and 5 and 12 weeks. Theoretical outcomes are the mediating role of self-reported perceived autonomy support, autonomous and controlled motivations, perceived competence, and barrier self-efficacy on steps per day. Clinical outcomes are quality of life, smoking, medication adherence, secondary prevention program attendance, health care utilization, and angina frequency. The potential moderating role of sex will also be explored. Analysis of covariance models will be used with covariates such as sex, age, fatigue, and depression symptoms. Allocation sequence is concealed, and blinding will be implemented during data analysis.

**Results:**

Recruitment started March 30, 2016. Data analysis is planned for November 2017.

**Conclusions:**

Finding alternative interventions aimed at increasing the adoption of health behavior changes such as physical activity in the secondary prevention of ACS is clearly needed. Our RCT is expected to help support the potential efficacy of a fully automated, Web-based tailored nursing intervention on the objective outcome of steps per day in an ACS population. If this RCT is successful, and after its implementation as part of usual care, TAVIE en m@rche could help improve the health of ACS patients at large.

**Trial Registration:**

ClinicalTrials.gov NCT02617641; https://clinicaltrials.gov/ct2/show/NCT02617641 (Archived by WebCite at http://www.webcitation.org/6pNNGndRa)

## Introduction

Acute coronary syndromes (ACS) are among the leading causes of coronary artery disease mortality and are among the top reasons for health care utilization in North America [1-3] and worldwide [4]. Physical activity is one behavior associated with several health benefits in ACS patients, including reduced mortality and health care utilization. Accumulating an equivalent of 150 minutes per week of moderate intensity physical activity is associated with reduced all-cause [5-7] and cardiac mortality risk [7] compared with lower levels of physical activity. Evidence from cohort data suggests that all-cause mortality risk can be reduced by accumulating half of the recommendation compared with zero minutes, and further reductions are obtained as physical activity increases [8], which may also be applicable to ACS populations. Other health benefits of increased physical activity in ACS include improved quality of life [9], reduced cardiac risk factors such as dyslipidemia and hypertension, and reduced health care utilization such as hospitalizations [10]. Moreover, positive change in one health behavior, such as an increase in physical activity, may increase overall confidence and serve as a gateway to changing other health behaviors [11], such as increased smoking cessation, healthy diet, medication adherence, or attendance in a cardiac secondary prevention program. Therefore, these multiple health benefits place increased physical activity as a cornerstone in the secondary prevention of ACS [10]. Despite these benefits, between 40% and 60% of patients were insufficiently active after an ACS event [5,6,12].

Increased physical activity is promoted in traditional secondary prevention programs that consist of face-to-face or phone health behavior change counseling, which may range from brief to intensive counseling, and most include supervised exercise in affiliated hospital settings [13]. However, only 22%-30% of cardiac patients attend face-to-face secondary prevention programs [14,15]. Barriers include the difficulty of accessing these programs among those living in remote locations where secondary prevention programs are not offered, traveling to meetings, or reaching those who lack motivation or are unwilling to participate in these programs. Therefore, alternative ways of delivering these programs are being examined in research, including use of the Web [13].

Web-based interventions aimed at improving health behaviors have been tested mostly in general adult populations. These interventions include modes of delivery such as online text, videos, and discussion forums, and include other modes complementary to websites such as email and text message [16]. A meta-analysis of randomized controlled trials (RCTs) and quasi-experimental studies in mainly general adult populations or adults with cardiac risk factors found a significantly greater effect on physical activity outcomes in Web-based interventions compared with usual care control groups that were not Web-based (*d*=0.14, *P*<.001) [17]. Although intervention effects were small, a greater effect was found in studies that included only insufficiently active participants compared with those that included any level of physical activity (*d*=0.37 vs 0.12, respectively, *P*<.05) [17].

Web-based tailored interventions are expected to increase the relevancy of and attention to the information delivered, which in turn is expected to improve effects on health behavior change [18,19]. Tailoring can be static, such that tailored messages are provided based on a single baseline assessment, or dynamic, such that tailored messages are provided based on multiple assessments from baseline to follow-up [20]. Although a meta-analysis of RCTs and quasi-experimental studies in mainly general adult populations or adults with cardiac risk factors found no differences between tailored versus non-tailored interventions on physical activity outcomes, the authors found a significant effect in favor of tailoring (static or dynamic) on smoking cessation and healthy diet outcomes [21]. Therefore, increased physical activity in Web-based interventions may not depend only on tailoring. Perhaps the combination of components within Web-based tailored interventions matters, such as the variables on which tailoring was based (eg, motivation and confidence), modes of delivery used (eg, combining online text, videos, email, and others), level of intervention intensity delivered, and target population characteristics. Therefore, further research is needed to test innovative combinations of these components in tailored interventions to influence greater increases in physical activity.

In ACS patients, a Cochrane review found some evidence in eight RCTs to support the effect on increased physical activity outcomes in favor of Web-based interventions (tailored or not) compared with usual care [22]. However, heterogeneity between these RCTs prevented a meta-analysis on physical activity outcomes [22]. Among these eight RCTs, one was a pilot [23], two were not powered on physical activity outcomes [24,25], and one was powered on a self-reported physical activity outcome, but results were limited by the majority of participants dropping out [26]. Only four RCTs were full-sized and powered on objective physical activity outcomes [27-30], among which, two tested tailored interventions [27,28]. Both found significantly greater levels in the primary outcome of steps per day in favor of the tailored experimental groups [27,28]. These data suggest that in ACS populations, the effects of tailored interventions on steps per day outcomes are promising.

The other two RCTs tested nontailored interventions measuring the primary outcome of exercise capacity compared with usual care [29,30]. One RCT found a significantly greater increase in a proxy outcome of exercise capacity, maximal time on treadmill, in favor of the experimental group [29]. In contrast, the other RCT found no difference between groups in treadmill-measured peak oxygen uptake, despite finding significantly greater increases in a subjective secondary outcome of self-reported physical activity in favor of the experimental group [30]. Considering these four RCTs, the content of the Web-based interventions tested were sufficient to increase steps per day [27,28] and maximal time on treadmill [29], but the exercise intensity was insufficient to increase peak oxygen uptake [30]. No RCTs tested Web-based interventions, with or without tailoring, in ACS patients performing insufficient physical activity. The paucity of strong evidence highlights the need for future full-sized RCTs testing Web-based tailored interventions on objective physical activity outcomes in ACS populations.

### Theoretical Framework

We designed the fully automated, Web-based tailored nursing intervention TAVIE en m@rche in French. “TA VIE” means *your life*, and “en marche” means *walking* as the intervention is focused on increasing walking behavior in one’s daily life after an ACS-related hospitalization. The tailored content of TAVIE en m@rche is presented to participants by prerecorded videos of a nurse. We used Strengths-Based Nursing Care (SBNC) integrated with Self-Determination Theory (SDT) as the intervention’s theoretical framework. SBNC describes an orientation to nursing practice or a “way of being” that is manifested through person-centered, holistic, knowledgeable, and compassionate nursing care [31]. SBNC is driven by eight values that focus on “understanding the whole, … and understanding how strengths and weaknesses interact to promote health, and healing” (p. 120) [31]: (1) health and healing refers to creating and restoring persons’ sense of wholeness in all domains of human functioning, (2) uniqueness of the person refers to understanding unique experiences and strengths, (3) holism and embodiment refers to understanding the complexities underlying the relationships among the mind, brain, and other body systems, (4) objective/subjective reality and created meaning refers to understanding along with objective observations, subjective realities through created meanings of persons’ experiences, (5) self-determination refers to respecting persons’ right to a life grounded in volition and free will, (6) person and environment are integral refers to understanding how persons’ environments influence health and healing, (7) learning, readiness, and timing refers to being sensitive to readiness and timing when engaging patients in an active learning or change process, and (8) collaborative partnership between nurse and person refers to both nurse and patient sharing knowledge and strategies that foster health and healing.

Self-determination, one of the eight SBNC values, is particularly relevant in nursing care, and in human motivation to adopt health behavior changes. This value was drawn from literature on self-determination including past works on the SDT of human motivation [32]. Empirical work in SDT applied in health care settings has presented two models [33]. The first model suggests that improvements in physical and mental health can be explained by the satisfaction of the psychological needs of autonomy, competence, and relatedness [33]. However, our Web-based intervention that has a minimal focus on encouraging social support from others may not be powerful enough to influence the construct of relatedness, which refers to the “feeling of being respected, understood, and cared for by others” (p. 327 [33]), such as exercise companions. Therefore, the second model that excludes the construct of relatedness [33] was retained. This model suggests that improvements in health behavior can be explained by improvements in three SDT constructs: increased perceived autonomy support, improved self-determined motivation (decreased controlled vs increased autonomous motivations), and increased perceived competence [33,34]. Perceived autonomy support refers to the perception that during an intervention or interaction with a significant other, choices were provided, rationale was offered, and acknowledgement or empathy was expressed [33]. Controlled motivation refers to actual or future behavior change that is imposed by others or that is motivated out of a sense of guilt and shame in the presence of failure in change [33]. Autonomous motivation refers to actual or future behavior change that is volitional, aligned with one’s goals and values, or motivated by sheer enjoyment [33]. Perceived competence, similar to self-efficacy [34], refers to the degree of confidence in one’s capability in achieving a health behavior change goal [33]. From the cardiac literature, barrier self-efficacy refers to degree of confidence in overcoming barriers towards health behavior change [35]. A systematic review found that the relationships between these SDT constructs and physical activity outcomes were well supported [36], suggesting that interventions that are efficacious at influencing positive changes in SDT constructs may also influence improvements in physical activity outcomes.

SDT is a novel approach to theoretical grounding in the Web-based physical activity literature as no studies using SDT were found in past meta-analyses in either general adult populations [17] or ACS patients [22]. However, we found three full-sized RCTs testing the effect of Web- and SDT-based interventions in general adult populations that were powered on self-reported physical activity outcomes [37,38] or a composite outcome that included physical activity [39]. Among the two RCTs powered on a self-reported physical activity outcome, the most recent found a significant increase of 71 minutes in weekly moderate to vigorous physical activity at 12 months in favor of the SDT-based intervention compared with a waitlist control [37]. In this RCT, the SDT-based intervention consisted of tailored messages delivered in text format and nontailored information (motivational and educational) delivered by videos of a physical activity expert. In the other RCT, the authors did not report effects on physical activity outcomes in their conference abstract [38]. In the RCT powered on a composite outcome of self-reported weight, diet, smoking, and physical activity, the authors reported no effect on the physical activity outcome, which was possibly due to a lack of intervention utilization because too many choices were given in intervention intensities and modes of delivery in the experimental group [39]. Therefore, the Web- and SDT-based intervention literature is sparse [37-39], despite the solid evidence supporting the positive associations between SDT constructs and physical activity outcomes [36]. To our knowledge, no RCT has tested a Web- and SDT-based intervention on physical activity outcomes in ACS patients whether sufficiently active or not. In addition, an innovation not yet examined in the Web-based ACS literature is the use of fully automated videos in tailored interventions. Use of videos could better convey the nurses’ strengths-based “way of being” because patients can view and listen to the nurse who presents tailored motivational and educational information instead of reading this same information in text format.

### Study Aim and Hypotheses

The aim of this RCT is to evaluate in insufficiently active ACS patients, the effect of a fully automated, Web-based tailored nursing intervention (TAVIE en m@rche) on increased steps per day. Our primary hypothesis is that ACS patients in the experimental group receiving TAVIE en m@rche compared with the control group receiving hyperlinks to publicly available websites will demonstrate a greater increase in change in steps per day between randomization and 12 weeks (H1). Secondary hypotheses are a greater increase in change in steps per day between randomization and 5 weeks (H2), and in energy expenditure for walking and moderate to vigorous physical activity between randomization and 5 weeks, and randomization and 12 weeks (H3 to H6).

We are interested in assessing if the change in SDT variables immediately postintervention at 5 weeks will explain the hypothesized increase in steps per day at 12 weeks. Therefore, we will explore the mediating role of the SDT constructs (perceived autonomy support, controlled and autonomous motivations, perceived competence) and barrier self-efficacy on the effect of TAVIE en m@rche on increased steps per day at 12 weeks (H7 to H11 respectively).

We will also explore the effect of TAVIE en m@rche at 12 weeks on improved quality of life (global, emotional, physical, and social), smoking, medication adherence, secondary prevention program attendance, emergency visits, and hospitalizations (H12 to H20). The potential moderating role of sex on the effect of TAVIE en m@rche on steps per day at 12 weeks will also be explored (H21). Finally, we expect no adverse effect, which is represented by an equal level of angina symptom frequency at 12 weeks in both groups (H22).

## Methods

This section is presented according to the SPIRIT 2013 statement in defining standard protocol items for clinical trials [40]. The completed CONSORT EHEALTH checklist [16] is found in Multimedia Appendix 1.

### Study Design and Settings

The study design is a two-group parallel multicenter RCT testing the effect of an experimental group that is receiving access to a 4-week Web-based tailored nursing intervention (TAVIE en m@rche) and a brief “booster” at 8 weeks, compared with a control group that is receiving access to hyperlinks of publicly available websites on increased steps per day. Study settings are at four major teaching hospital centers in Montreal, Canada.

### Eligibility Criteria

Patients are eligible if they are home the third week after an ACS-related hospitalization, have no serious medical condition that would impede adhering to moderate intensity physical activity, report access in any location to a computer with a USB port that is connected to the Internet, and report the ability to read and speak French. Patients are ineligible if they self-report sufficient physical activity during 6 months prior to the hospitalization where they are recruited (ie, performed at least 150 minutes [30 minutes 5 days a week] of moderate intensity physical activity per week or at least 75 minutes [25 minutes 3 days per week] of vigorous intensity physical activity per week), have documented New York Heart Association Class III-IV heart failure, or reported planned involvement in intensive regular clinical follow-up (eg, an outpatient heart failure clinic) during TAVIE en m@rche. One hospital center asked that those who are eligible for participation in their onsite secondary prevention program (ie, new diagnosis of ACS and age <75 years) be ineligible for study participation to avoid delivery of parallel secondary prevention interventions at that center.

### Interventions

Both groups receive usual care from hospital entry to return home. At all four recruitment centers, from hospital entry to hospital discharge, usual care consists of brief counseling by hospital staff on discharge issues such as new medications and their side effects and on health behavior changes such as progressively increasing physical activity at home. Printed materials are provided as teaching aids to complement the brief counseling. As well, patients receive referrals to onsite or community-based secondary prevention programs.

After hospitalization, all centers offer secondary prevention programs, but at varying doses. All offer an educational group program on the topic of cardiac risk factors and health behaviors aimed at reducing these risk factors, but the number of sessions varies between one and eight across the four centers. Also, three of the four centers offer onsite supervised exercise programs, but the number of sessions vary between one to three times per week. Two of the programs are 12 weeks in duration, and the other lasts 1 year.

#### Control Group

The control group receives a list of four hyperlinks on a unique webpage of available online information that is Canadian, French, and that included information on walking. Three major Canadian nonprofit or public organizations are included: Montreal Heart Institute, Heart and Stroke Foundation, and Canadian Society for Exercise Physiology. In addition, because key recommendations on walking post‒ACS-related hospitalization were derived from a patient education booklet published by the Montreal Heart Institute that is available online, the walking program in this booklet is also included in the list of hyperlinks. All websites provide information in text format without the use of videos.

#### Experimental Group

The experimental group receives access to TAVIE en m@rche. The central feature of TAVIE en m@rche is the prerecorded videos of a nurse (see Multimedia Appendix 2) who presents the fully automated tailored intervention content delivered according to patients’ baseline assessments of autonomous motivation, perceived competence, and walking behavior. Other modes of delivery include online text that appears beside the videos to allow simultaneous reading of the video’s content, and downloadable PDF files referred to by the nurse. Access to TAVIE en m@rche starts at randomization between the fourth and fifth week after hospitalization, which depends on when the baseline online assessment is submitted. The suggested completion time of the intervention is 4 weeks but access to the intervention ends at 11 weeks postrandomization. An additional brief “booster” is added at 8 weeks postrandomization. We estimate about 60-75 minutes is sufficient to complete the intervention. TAVIE en m@rche consists of 73 videos, each lasting on average nearly 1 minute with most (n=68) lasting less than 2 minutes. The TAVIE platform has simple webpage layouts and is easy to navigate [41].

The intervention goal is to encourage a progressive increase in walking behavior, up to the recommended 150 minutes per week at moderate intensity, which is determined by an adapted version of the Borg Rating of Perceived Exertion [42]. This walking level is recommended to all patients at discharge for an ACS-related hospitalization by their treating physician unless a contraindication is present such as comorbid physical condition or an environmental constraint. Such patients are ineligible for study participation.

The intervention is based on a theoretical framework that integrates SBNC with SDT. SBNC focuses on nursing values such as fostering a collaborative partnership with the person, supporting the person’s self-determination in their decisions and actions, and working with the person’s strengths in the aim of achieving health and healing. The SDT on human motivation specifies theoretical constructs for physical activity to be targeted by the intervention strategies and to drive the tailoring process. The intervention strategies are specifically targeted toward increasing self-reported perceived autonomy support, autonomous motivation, and/or confidence (combined perceived competence and barrier self-efficacy).

The appeal of using videos as the main mode of delivery, rather than text-only format, is the greater ability to convey the strengths-based nursing way of being that is manifested in part by nonverbal behaviors such as tone of voice (eg, energetic vs neutral) and body language (eg, smiling vs a sincere nonjudgmental expression), and by verbal behaviors (ie, the nurse’s script). This script, consistent with both SBNC and SDT, drawn from our past literature review [43], followed five global strategies: being collaborative, being strengths-focused, providing choice, offering rationale, and expressing empathy. These global strategies can be thought of as the fabric in which the entire intervention content is interwoven. As such, we expect that the use of videos instead of text-only format will be more interesting and motivating to participants because the SBNC way of being will be better conveyed.

The intervention consists of four specific strategies targeting increasing perceived autonomy support, autonomous motivation, and/or confidence aimed at increasing walking behavior: Strategy (1) Providing information and feedback to build motivation and confidence; Strategy (2) Exploring reasons to build motivation; Strategy (3) Exploring personal strengths to build confidence; and Strategy (4) Developing an action plan to build and consolidate motivation and confidence. These strategies are operationalized by 19 behavior change techniques. The terminologies of those 19 techniques were made consistent with those of the CALO-RE taxonomy [44] (Table 1). These behavior change techniques were drawn from three main literary sources: (1) SDT-based physical activity face-to-face or Web-based interventions and Motivational Interviewing due to its consistency with SDT [45], (2) facilitators of physical activity such as improved cardiac health and quality of life, and barriers of physical activity such as lack of time and the presence of fatigue and depressive symptoms found in cardiac patients, and (3) two patient education booklets on the secondary prevention of ACS [42,46]. The content validity of comparing intervention strategies (global and specific) with the theoretical background was done by the Université de Montréal scientific PhD jury of the first author. One cardiac nurse reviewed the operationalization of the entire intervention, and two clinical kinesiologists in secondary prevention reviewed the operationalized information on walking.

**Table 1:** Specific strategies, intermediate intervention goals, behavior change techniques, and targeted SDT variables.

Specific strategy	Intermediate intervention goal	Behavior change technique	Targeted SDT variable^a^
1. Providing information and feedback on walking behavior	To help patients build or consolidate motivation and confidence to increase walking behavior or maintain sufficient walking behavior	1.1 Provide information on consequences of behavior in general by providing information on potential advantages of physical activity through walking	Perceived autonomy support from the intervention Autonomous motivation
		1.2 Provide instruction on how to perform the behavior of attaining the recommended minutes per week of physical activity through walking	Perceived autonomy support from the intervention Confidence
		1.3 Provide feedback on performance tailored to minutes per week of walking in the past 7 days	Perceived autonomy support from the intervention Confidence
2. Exploring reasons to increase walking behavior	To help patients build motivation to increase walking behavior	2.1 Motivational interviewing, asking evocative questions to explore advantages of increasing walking behavior, and to explore goals and values^c^	Perceived autonomy support from the intervention Autonomous motivation
		2.2 Motivational interviewing, sharing a list of potential reasons to increase walking behavior^c^	Perceived autonomy support from the intervention Autonomous motivation
3. Exploring strengths	To help patients build confidence to increase walking behavior	3.1 Motivational interviewing, asking evocative questions to explore strengths^c^	Perceived autonomy support from the intervention Confidence
		3.2 Motivational interviewing, sharing a list of potential strengths^c^	Perceived autonomy support from the intervention Confidence
4. Developing an action plan	To help patients consolidate their motivation and confidence to increase walking behavior or maintain sufficient walking behavior	4.1 Provide instruction on how to perform the behavior of perceived exercise exertion assessment and planning walking in four steps	Perceived autonomy support from the intervention Confidence
		4.2 Teach to use prompts/cues using flash card of perceived exertion and the four steps	Perceived autonomy support from the intervention Confidence
		4.3 Goal setting using SMART goals	Perceived autonomy support from the intervention Confidence
		4.4 Provide information on consequences of behavior in general by providing information on potential advantages of walking, and how to make walking enjoyable	Perceived autonomy support from the intervention Autonomous motivation
		4.5 Teach to use prompts/cues using flash card of SMART goals and reasons for walking	Perceived autonomy support from the intervention Autonomous motivation Confidence
		4.6 Prompt self-monitoring of behavior of SMART goals	Perceived autonomy support from the intervention Autonomous motivation^b^ Confidence
		4.7 Provide information on where and when, and instruction on how to perform the behavior using practical tips to increase walking behavior or to maintain sufficient walking behavior	Perceived autonomy support from the intervention Confidence
		4.8 Prompt review of the identification of behavioral goals (SMART goals, and reasons for walking)	Perceived autonomy support from the intervention Autonomous motivation Confidence
		4.9 Barrier identification/problem solving	Perceived autonomy support from the intervention Autonomous motivation^b^ Confidence
		4.10 Plan social support to elicit support from significant others in the attainment of increasing walking behavior or maintaining sufficient walking behavior	Perceived autonomy support from the intervention Perceived autonomy support from a significant other
		4.11 Provide an example of action planning	Perceived autonomy support from the intervention Autonomous motivation^b^ Confidence
		4.12 Provide feedback on performance (action plan and walking behavior)	Perceived autonomy support from the intervention Confidence

^a^Perceived autonomy support from the intervention is targeted throughout because the global strategies (Being Collaborative, Being Strengths-Focused, Providing Choices, Offering Rationale, and Expressing Empathy), which are consistent with both SBNC and SDT, are integrated within each specific strategy.

^b^Autonomous motivation targeted 4.6 in the enjoyment in monitoring the accomplishments of a SMART goal; 4.9 in two barriers: (1) not having enough time to walk, and (2) having no reason to walk; and 4.11 in the example of reasons for increasing walking behavior within an action plan.

^c^Motivational Interviewing is reported here as behavior change techniques consistent with the CALO-RE taxonomy and is limited to open-ended questions consistent with Motivational Interviewing, without the back-and-forth aspect of face-to-face counseling found in an interview.

**Figure 1:**
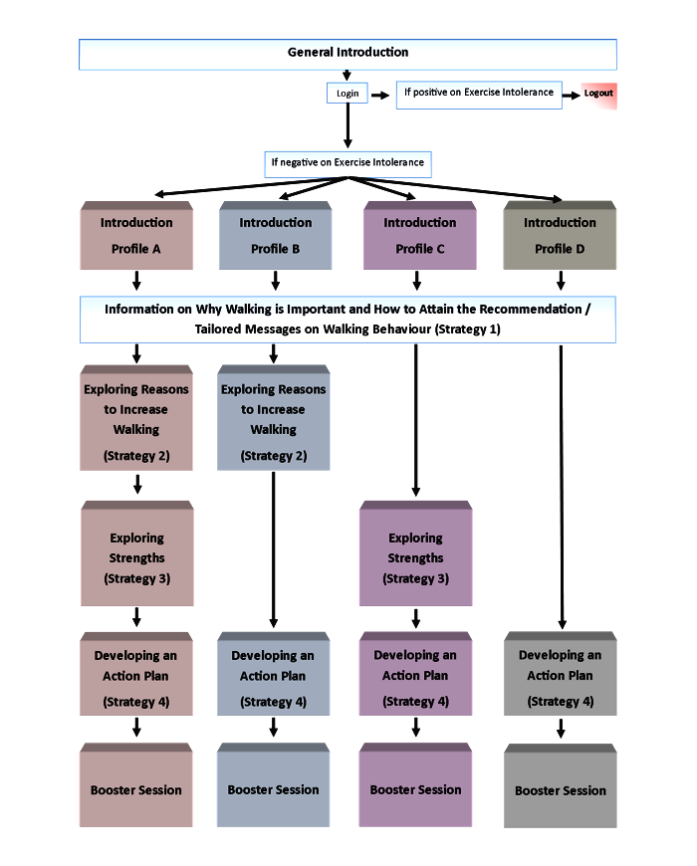
Schema of the intervention’s general and per profile introductions, and the four specific intervention strategies.

The strategies are conveyed through a set of videos that build toward participants’ commitment to developing their own action plan. The order of the strategies is determined by the primary static tailoring method that is driven by participants’ baseline self-reported autonomous motivation (low vs high), confidence (low vs high), and walking behavior, which resulted in four tailored profiles: A, B, C, and D (Figure 1). Profiles A, B, and C are assigned from scores that are below the recommended 150 minutes per week of walking. Profile A receives Strategies 1 (information), 2 (reasons), 3 (strengths), and 4 (action plan) because this profile is low in motivation and confidence. Profile B receives Strategies 1 (information), 2 (reasons), and 4 (action plan) because this profile is low in motivation. Profile C receives Strategies 1 (information), 3 (strengths), and 4 (action plan) because this profile is low in confidence. Profile D receives Strategies 1 (information), and 4 (action plan) because this profile is high in motivation and confidence. In addition, participants who attained the recommended minutes per week of walking between hospital discharge and baseline receive Profile D.

Secondary methods are the use of tailored messages based on “yes” versus “no” responses to questions after intervention login on identifying symptoms of exercise intolerance in the past 7 days (Introduction). Participants who respond “yes” to having identified symptoms of exercise intolerance are provided an onscreen video message asking them to not initiate the intervention, to consult a free 24-hour province-wide phone service for general health problems if the symptoms are nonurgent, or to call 9-1-1 or go to the emergency department if the symptoms are urgent, and then to log out of the intervention. Two weeks later, participants are asked to log in to the intervention, and only if no symptoms of exercise intolerance are identified by the participant, they are invited to continue the intervention. Static tailored messages on walking behavior (ie, feedback on performance) are also provided to participants in all four profiles (Strategy 1 information) based on their responses of walking behavior assessed only at baseline. Other tailored messages based on “yes” versus “no” responses to questions after intervention login pertain to the identification of personal reasons for walking (Strategy 2 reasons), personal strengths (Strategy 3 strengths), personal goals that are SMART (Specific, Measurable, Attainable, Realistic, and within a Timeframe) (Strategy 4 action plan), social support, and solutions to barriers (Strategy 4 action plan).

### Timeline and Procedures

The study duration is 16-17 weeks, from hospitalization (-T2) to the last assessment at 12 weeks postrandomization (T3). We estimate that 4 hours in the experimental group and 2.5 hours in the control group are needed to participate in the study, which includes time spent in either experimental or control interventions, and the completion of the questionnaires (Table 2).

**Table 2:** Schedule of enrollment, interventions, and assessments.

				Minutes per patient	Recruitment	Baseline	Randomization	Interventions	Follow-up 5 wks	Follow-up 12 wks
Activity			Items		-T2	-T1	T0	T1	T2	T3
**Eligibility screening**										
	Patient lists			N/A	x					
	Inclusion/exclusion interview			~10	x					
	Screening log			N/A	x					
Consent and signing				~30	x					
Instruction to wear accelerometer and complete questionnaires				~10		x				
Randomization and allocation to group				~1			x			
Access to experimental or control group interventions				~60-75				x		
Documentation				N/A	x	x	x	x	x	x
**Assessments in-hospital**										
	Sociodemographic data and depression questionnaire		19	~15	x					
	Give and explain accelerometer wear			~10	x					
	Clinical data (eg, history, tests, events, and cardiac risk factors)			N/A	x					
**Assessments at home**										
	Intervention adherence			N/A				x		
	Primary outcome (X) steps/day			N/A		X			x	X
	**Questionnaires**			~30-45						
		Self-reported physical activity and location of accelerometer wear	7			x			x	x
		Perceived autonomy support of significant other	6						x	
		Perceived autonomy support of websites	6						x	
		Autonomous and controlled motivations	12			x			x	
		Perceived competence	4			x			x	
		Barrier self-efficacy	8			x			x	
		Quality of life	27			x				x
		Smoking status	1			x				x
		Medication adherence	4			x				x
		Secondary prevention program enrollment	2							x
		Angina frequency	2			x				x
		Fatigue	7			x				
	**Clinical data**									
		Emergency visits and hospitalizations		N/A						x

**Figure 2:**
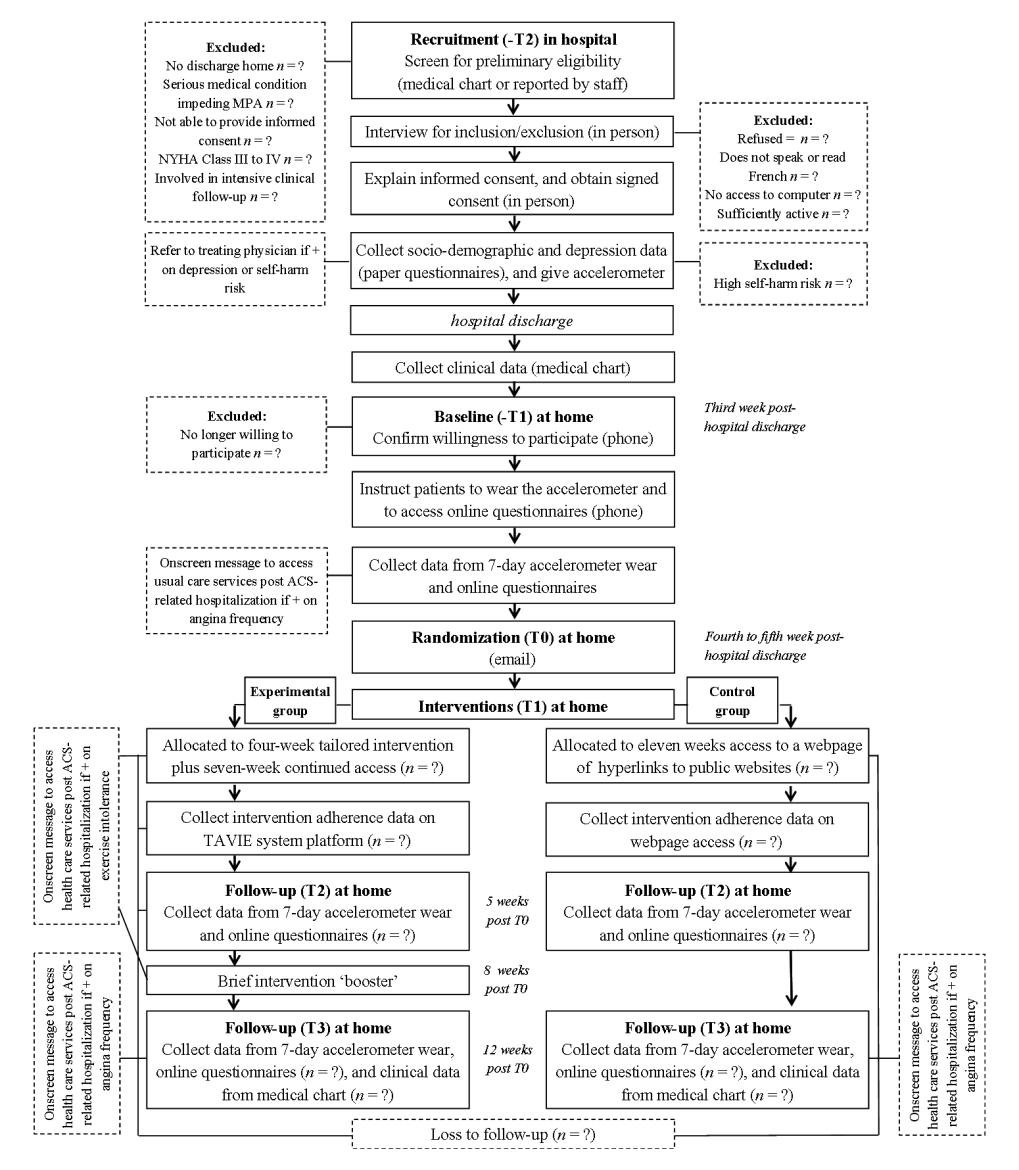
Flow of participants.

Recruitment (-T2) takes place in-hospital. Potential participants are identified through patient lists during hospitalization, and we then proceed with preliminary eligibility screening using patients’ medical charts (Figure 2). Eligibility screening, rather than being based on a 24-hour and 7-day a week schedule, is based on the recruiters’ irregular schedules, which vary depending on their availability to present at one of the four sites or on other constraints such as work (academic or other) unrelated to recruiting. When potential participants are approached in hospital, eligibility is confirmed (ie, inclusion/exclusion based on in-person interview), and the study protocol is explained. After signed consent is obtained, self-administered paper questionnaires on sociodemographic data and depressive symptoms are completed. Participants are then given an accelerometer and an open prepaid envelope to use when sending it back to the researcher at the end of the study. After hospital discharge, clinical data are collected from the medical chart.

Baseline (-T1) is at the beginning of the third week posthospital discharge. In conjunction with an email, participants are contacted by phone to confirm their willingness to participate. This email includes a hyperlink to download and install the software into the participants’ computers, allowing the accelerometer data to be synced to the company’s server. Thereafter, the data can then be downloaded into the researcher’s computer. During the same phone call, participants are instructed to wear the accelerometer daily for 7 days from awakening to bedtime. After 7 days of accelerometer wear, a second email is sent that includes a hyperlink to access the first online questionnaire. Although we expect most participants to complete the baseline accelerometer wear followed by the online questionnaire within 1 week, a maximum window of 2 weeks is allowed to complete baseline assessments.

Randomization (T0) and allocation to the experimental or control groups occur upon submission of the baseline questionnaire at the fourth or fifth week posthospital discharge, allowing participants the window of 2 weeks to complete baseline assessments. Each participant receives, by automated email from the TAVIE platform, access to either TAVIE en m@rche or the control group involving publicly available websites.

Interventions (T1) start immediately after allocation to the experimental or control groups. Follow-ups at 5 weeks (T2) and 12 weeks (T3) postrandomization are planned. At both follow-ups, participants in both groups (experimental and control) are sent an email with instructions to wear the accelerometer for 7 days. If participants accept, a brief text message is sent to remind them to read the email. After 7 days of accelerometer wear, a second email is sent to complete the online questionnaires. In addition, at the end of data collection, participants are instructed to return the accelerometer by mail via the prepaid envelope provided. During participation, the first author is available by phone and email to resolve technical difficulties in accessing the intervention or questionnaires.

### Sample Size Calculation

To detect a difference in change between randomization and 12 weeks of 1500 steps per day (SD 2824) in favor of the experimental group compared with the control group, a total of 148 participants (n=74 participants per group [57 plus 17 for attrition], and n=37 per recruitment center) is needed, given a two-sided 5% significance level, power of 80%, and an expected attrition of 23%. The 1500 steps per day is an approximation of half of the recommended daily minutes of moderate intensity physical activity [47]. The attrition of 23% was reported in a meta-analysis of Web-based interventions, in which the experimental groups had an average intervention duration of 13 weeks [17]. The SD of 2824 steps per day was estimated using data found in an RCT of a counseling intervention in ACS patients in Quebec [48].

### Randomization and Allocation

Randomization is planned by an offsite coordinating center. It is stratified by study center to help protect against between group imbalances if recruitment differs in one or more study centers [49]. Per stratum, random numbers are given for each assignment. Random assignments follow a 1:1 allocation using random block sizes determined by the coordinating center to minimize the chance of group imbalances [49].

The assignment sent by the coordinating center in electronic list (.xls) format was uploaded in the TAVIE platform. The allocation sequence is concealed. Upon submission of the baseline questionnaire, the TAVIE platform sends an automated email of the assignment to the participant. This email includes the hyperlink and password to access the experimental TAVIE en m@rche or to receive a different hyperlink to access the control of publicly available websites.

### Blinding

Care providers during hospitalization (treating physicians, nurses, and others) are blinded to group assignment because randomization occurs posthospitalization. The first author must know of the assignment after it is revealed, in order to manage the emails sent to participants in either the experimental or control group. The outcome data completed online are anonymized allowing blinding to group assignment. Participants are not blinded to group assignment because they consent to randomly receiving one of two website hyperlinks. Although participants are not informed as to which website is experimental versus control, they are informed that one website takes about 60-75 minutes to complete (ie, experimental), and the other website takes an undetermined number of minutes to complete (ie, control).

### Outcomes

#### Primary Outcome of Steps per Day

The primary outcome is change in steps per day measured by an accelerometer between randomization and 12 weeks. We chose 12 weeks as the primary endpoint rather than 5 weeks to assess the persistence of steps performed beyond the end of the intervention’s 4-week period. The accelerometer step count data are concealed, uploaded wirelessly to a server [50], and downloaded in the first author’s computer. Similar to step counts measured by a previously validated pedometer, step counts measured by the accelerometer worn on a shoe had less than 2% error compared with observed step counts measured by hand tally counter [50]. Participants are instructed to clip the accelerometer on one of their shoes during waking hours. If they are not wearing shoes, they are then instructed to clip it at their waistline clothing (belt or pants) as recommended by the manufacturer.

The steps per day mean will be estimated using ≥3 valid step-days within the 7-day wear period, which is an accepted norm in adult populations [51]. A valid step-day will be determined by a wear time of ≥10 hours per day [51]. Fewer than 3 valid step-days will be treated as missing data.

#### Secondary Outcomes of Steps per Day and Energy Expenditure

Secondary outcomes are change in steps per day measured by an accelerometer between randomization and 5 weeks, and in self-reported energy expenditure for walking, and for moderate to vigorous physical activity measured by the French short version International Physical Activity Questionnaire (IPAQ) [52] between randomization, and 5 and 12 weeks. For self-reported energy expenditure, we retained six of the seven items that provided a single continuous score of Metabolic Equivalent of Task (METs)-minutes per week in the last 7 days. The score of energy expenditure for walking is the product of days performed in walking, minutes performed per day, and 3.3 METs. The score of energy expenditure for moderate to vigorous physical activity is the sum of two products: the product of days performed in moderate intensity physical activity (eg, carrying light loads or bicycling at a regular pace), minutes performed per day, and 4.0 METs; and the product of days performed in vigorous intensity physical activity (eg, heavy lifting, or fast bicycling), minutes performed per day, and 8.0 METs. International studies found that the reliability (test-retest) and criterion validity (self-report vs accelerometer data) of the IPAQ generally score around .80 (reliability) and .30 (criterion validity), which is comparable with psychometrics of other self-report physical activity questionnaires [53].

#### Outcomes of Theoretical Variables

Two outcomes assessed only at 5 weeks for Perceived autonomy support (PAS) were drawn from a French version of the Important Other Climate Questionnaire: (1) from a significant other (PAS-SO) and (2) from the intervention (PAS-WEB). These measures assess autonomy support felt from a significant other (PAS-SO) and from either research website visited (PAS-WEB). The two scores are the mean of responses of 6 items for each PAS (significant other [SO] vs intervention [WEB]) rated between “not at all true” (1) and “very true” (7). Higher scores represent greater levels of PAS. Reported Cronbach alphas across three assessments were between .86 and .89 [54].

Self-determined motivation is assessed at baseline and 5 weeks by the French version of the Treatment Self-Regulation Questionnaire. This measure assesses reasons to attain the recommendation of walking 150 minutes per week. We retained the 12 items that assess controlled motivation (6 items) and autonomous motivation (6 items). The two scores are the mean of responses of 6 items for each motivation (controlled vs autonomous) rated between “not at all true” (1) and “very true” (7). Higher scores represent greater levels of controlled and autonomous motivation. Reported Cronbach alphas across four populations were between .73 and .91 for controlled motivation, and between .85 and .93 for autonomous motivation [55].

Perceived competence is assessed at baseline and 5 weeks by the French version Perceived Competence Scale [53]. This measure assesses confidence to attain the recommendation of walking 150 minutes per week. The score is the mean of responses of 4 items rated between “not true at all” (1) and “very true” (7). Higher scores represent greater levels of perceived competence. Reported Cronbach alphas across two assessments were .93 and .96 [54].

Barrier self-efficacy is assessed at baseline and 5 weeks by the French version Barrier Self-Efficacy Scale for cardiac patients [35]. This measure assesses confidence to walk for the recommended 150 minutes per week even if one or more of eight barriers listed are experienced. We retained 8 of the 9 items. The item removed referred to the barrier of experiencing angina or chest pain. Instead of overcoming this barrier, we expect that participants treat the pain and consult a health care professional if the pain is not relieved instead of continuing to increase their walking behavior. The score is the mean of responses of 8 items rated between “(0%) not at all confident” and “(100%) very confident.” Higher scores represent greater levels of barrier self-efficacy. A reported Cronbach alpha was .86 in the original 9-item scale [35].

#### Clinical Outcomes

Quality of life is assessed at baseline and 12 weeks by the French version MacNew Heart Disease Health-related Quality of Life Questionnaire for cardiac patients [28]. The 27 items assess, in the previous 2 weeks, global quality of life and its 3 subdimensions: emotional (14 items), physical (13 items), and social (13 items) [56]. Items include reverse scores and overlap across dimensions. The scores for global quality of life and each dimension are the mean of responses that range between 1 (poor quality of life), and 7 (high quality of life). Reported Cronbach alphas were .94, .94, .89, and .90 in global, emotional, physical, and social respectively [57].

Self-reported 7-day point prevalence smoking status, an accepted norm in assessing smoking status outcomes [58], is assessed at baseline and 12 weeks. The following question is used: “Have you smoked a cigarette, even a puff, in the past 7 days?” (p. 4 [59]), answered with “yes” (0), “no” (1), or “never smoked” (2). Point prevalence assessment had a sensitivity of 96.9% and specificity of 93.4% in detecting dichotomous smoking versus nonsmoking status compared with urinary cotinine [60].

Medication adherence is assessed at baseline and 12 weeks by the Self-Reported Morisky Medication Adherence Scale (MMAS-4) [61]. This measure assesses barriers to cardiac medication use in the previous 2 weeks such as forgetting to take them and stopping them because one feels well. The score is the sum of 4 items rated “no” (0) or “yes” (1) such that lower scores indicate better medication adherence. Scores are dichotomized between medium to low (1 to 4) and high (0). The MMAS-4 had a sensitivity and specificity of 81.0% and 44.0% respectively in predicting controlled blood pressure [61].

Secondary prevention program attendance is measured at 12 weeks by self-report rated by “no” (0) or “yes” (1) of at least one visit, since hospitalization, to a secondary prevention program that offers clinical follow-up with a health care professional for general health, medication adherence, healthy diet, smoking cessation, or exercise. No data on baseline attendance are collected because programs may start 4 weeks or later posthospitalization, which falls around the planned time of randomization.

Data for both emergency department visits and hospitalizations are collected from the medical records at 12 weeks at each study center. For each outcome, one or more emergency department visits or hospitalizations for any reason indicate a score of 1 and no emergency department visits or hospitalizations indicate a score of 0.

Angina frequency is assessed at baseline and 12 weeks by the angina frequency scale of the Seattle Angina Questionnaire. This measure assesses frequency of angina pain and nitroglycerin use that we changed from “in the past 4 weeks” to “in the past 2 weeks.” The score is the sum of responses of 2 items rated between “4 or more times per day” (1) and “none over the past 2 weeks” (6), which is then transformed to score between 0 (worst) and 100 (best). Lower scores represent greater angina frequency. A reported significant positive association was *r*=.31 between greater angina frequency and greater number of refills of sublingual nitroglycerin tablets in the previous year [62].

#### Sociodemographic and Clinical Data

At recruitment in hospital (-T2), sociodemographic and clinical data are collected. Nine items in a paper-based self-report questionnaire assess employment, education, marital status, and other demographics. Other data including medical history, diagnosis, laboratory tests, in-hospital events, cardiac risk factors, intermittent claudication, and documented referral to a secondary prevention program are collected from the medical chart after hospitalization. Also, depressive symptoms are assessed at recruitment (-T2) by the 9-item French version Patient Health Questionnaire (PHQ-9). The PHQ-9 administered at recruitment in hospital allowed us to refer participants with abnormal scores to the treating cardiologist. At baseline (-T1), fatigue is measured by the 7-item short form French version [63]. Based on our literature review prior to commencing our RCT, depression and fatigue were retained as potential covariates rather than outcome variables because of the uncertainty that Web-based interventions in ACS populations can decrease depression symptoms [28] and because it is unknown if such interventions can decrease fatigue as this variable has not been previously tested in the Web-based ACS literature.

#### Intervention Adherence

During the intervention (T1), intervention adherence data are collected. For TAVIE en m@rche, data are collected on the number of times videos and webpages are viewed and documents are downloaded. Time spent in the intervention will be estimated from these data. For the control group website, data on the number of website visits per person are collected by Google Analytics. Because the control group is provided a single webpage of hyperlinks of publicly available websites, collecting data from these websites is not possible.

### Statistical Methods

The Montreal Health Innovations Coordinating Center provided expertise for the statistical methods. Baseline characteristics will be compared using descriptive statistics to identify trends in group imbalances, and the analyses will be consistent with intention-to-treat principles, in which data at a given time point will not be excluded from the analysis [64]. Missing data will be examined and handled according to best practice in that field [65].

For the analyses of single continuous variables (eg, the primary outcome of change in steps per day), repeated measures analysis of covariance models will be used with covariates that include gender, age, diabetes, intermittent claudication, baseline smoking status, depression symptoms, and fatigue. For analyses of multiple continuous variables (eg, the secondary outcomes of change in walking and moderate to vigorous physical activity), repeated measures multivariate analysis of covariance models will be used with the same covariates as the above model. For single dichotomous variables with baseline values (eg, smoking status), sequential logistic regression models will be used. For single dichotomous variables without baseline values (eg, hospitalizations), chi-square models will be used. A mediation analysis will use a sequence of one-way analysis of variance models with Bonferroni adjustments, in which the alpha will be divided by the number of tests performed. Adjusted and unadjusted means or proportions in each group (experimental and control) will be provided along with a 95% confidence interval. No adjustments in *P* values will be made for the hypotheses on secondary and tertiary outcomes because these are aimed at supporting the primary hypothesis on steps per day rather than claiming intervention effect [66].

### Ethical Considerations

Ethics approval for this multicenter RCT was obtained from the Scientific and Ethics Committee of the Montreal Heart Institute Research Center (reference #MP-33-2015-1887). Procedures follow the mechanism of multicenter studies outlined by the Quebec Ministry of Health and Social Services [67].

We expect that the study population has no additional adverse effects in participating in this RCT because the recommendation for physical activity (ie, walking 150 minutes per week) is consistent with current cardiology practice. Also, past research found that cardiac patients can safely participate in physical activity at home [68,69]. As such, we hypothesize that angina frequency will be equivalent in both groups (experimental vs control).

## Results

This RCT is currently recruiting. Recruitment started March 30, 2016, and data analysis is planned for November 2017.

## Discussion

### Limitations

We aim to test in insufficiently active ACS patients, the effect of receiving a Web-based tailored nursing intervention (TAVIE en m@rche) on increasing steps per day compared with receiving hyperlinks to publicly available websites. There exist potential limitations in our RCT pertaining to outcomes, intervention, and generalizability.

#### Outcomes

First, although our primary outcome of steps per day was retained based on the literature showing the association between increased physical activity and reduced mortality in ACS patients [5-7], we did not plan mortality as primary or secondary outcomes. Trials that aim to improve physical activity outcomes usually establish eligibility criteria to select populations that are capable of attaining the amount of physical activity recommended in the intervention. As such, these populations have few comorbidities resulting in low serious adverse cardiac events or mortality. Two RCTs testing Web-based interventions in ACS patients measured an objective physical activity outcome (steps per day or exercise capacity) and reported serious adverse cardiac or mortality events requiring hospitalization per treatment group [28,29]. Reid et al. reported four hospitalisations for chest pain and no deaths in the experimental group, and six hospitalisations for chest pain, one for cardiac surgery and two deaths in the control group during 12 months of follow-up [28] Lear et al. reported three (9%) major cardiac events (e.g., revascularisation, stroke, and death) in the experimental group, and six (16%) in the control group during 16 months of follow-up [29]. These data suggest that fewer serious adverse cardiac or mortality events are found in favor of Web-based experimental groups, and too few events occur to plan mortality as a primary or secondary outcome within a feasible timeframe.

Second, for our secondary outcome of energy expenditure, we plan estimates from self-reported data instead of from accelerometer data. Accelerometers require the entry of participants’ weight in the devices to produce the estimates. However, we do not collect data on weight from participants at home before randomization (baseline [-T1]) because these data may be missing or unreliable from self-report or from participants’ own weighing scales.

Third, we planned a relatively short follow-up of 12 weeks for feasibility reasons as this RCT is part of a doctoral degree. A longer follow-up on steps per day and other health behavior changes in a future RCT testing TAVIE en m@rche could improve clinical relevance.

Fourth, it is possible that accelerometers are worn by or data from online questionnaires are entered by someone other than the study participant because the outcome data are completed by participants at home. Although we will treat this possibility when examining the outliers, which could reveal some data clearly out of range entered by a different respondent, there are no other provisions made for this limitation.

Fifth, there is a possibility of missing outcome data. Different scenarios for handling missing data will be followed according to best practice in that field [65] by a statistician who is part of an internationally recognized clinical trial reference center (Montreal Health Innovations Coordinating Center). The method of handling missing data will be reported in a future publication of the results.

#### Interventions

The platform is limited to using static tailoring rather than dynamic tailoring [20]. However, a recent meta-analysis found that dynamic tailoring has not improved effects on health behavior change outcomes compared with static tailoring [21].

#### Generalizability

Our sample will likely have similar characteristics as the four other RCTs testing a Web-based intervention using steps per day or another objective physical activity outcome in an ACS population [27-30]. Such populations have no important comorbidities or environmental constraints that would impede performance in moderate-intensity physical activity. ACS populations with important comorbidities are neither eligible to participate in our study nor eligible to receive the recommendation to gradually attain moderate intensity walking beginning the fourth or fifth week after hospitalization, as is recommended in TAVIE en m@rche. Therefore, the eligibility criteria for our RCT is comparable to the ACS population intended for TAVIE en m@rche. Another related cardiac population that could benefit from TAVIE en m@rche, after some minor modifications, are those with stable coronary artery disease, such as stable angina patients requiring elective percutaneous coronary intervention. However, our future results will not be generalizable in stable coronary artery disease populations.

### Conclusion

Alternative interventions aimed at increasing the adoption of health behavior changes in the secondary prevention of ACS are clearly needed. Our proposed intervention fills a gap in the literature because no RCT has tested a Web- and SDT-based tailored intervention using videos of a nurse on an objective physical activity outcome in insufficiently active ACS patients. Study strengths include the retained design, a full-scale RCT, which will confirm with confidence the effect of receiving TAVIE en m@rche on the objective primary outcome of steps per day in ACS patients. Also, the intervention’s theoretical framework and its operationalization enhance reproducibility. Finally, the framework allows the examination of theoretical processes, such as the SDT constructs, which may explain the intervention’s effects on the primary outcome. If this RCT is successful, and after its implementation as part of usual care, TAVIE en m@rche could help improve the health of ACS patients at large.

## Appendix

Multimedia Appendix 1 CONSORT-EHEALTH checklist V1.6.2 [16].

Multimedia Appendix 2 Screenshot of an experimental group webpage.

Multimedia Appendix 3 Peer review approval letter of the first author’s scientific committee jury of the Université de Montréal (French only).

## Abbreviations

ACS: acute coronary syndrome
IPAQ: International Physical Activity Questionnaire
MET: Metabolic Equivalent of Task
MMAS-4: Morisky Medication Adherence Scale
PAS: perceived autonomy support
PAS-SO: perceived autonomy support from a significant other
PAS-WEB: perceived autonomy support from the intervention
PHQ-9: Patient Health Questionnaire
RCT: randomized controlled trial
SBNC: Strengths-Based Nursing Care
SDT: Self-Determination Theory
