# Supplementary material for: Evaluation of a Web-Based Tailored Nursing Intervention (TAVIE en m@rche) Aimed at Increasing Walking After an Acute Coronary Syndrome: A Multicenter Randomized Controlled Trial Protocol
Source: JMIR Res Protoc. 2017 Apr 27;6(4):e64. doi: 10.2196/resprot.6430 (PMC5427251; doi:10.2196/resprot.6430)
Supplement: Multimedia Appendix 3 [file resprot_v6i4e64_app3.pdf]

Université de Montréal

Faculté des sciences infirmières

PROGRAMME CONJOINT DE DOCTORAT (SCIENCES INFIRMIERES)
APPROBATION SCIENTIFIQUE DU PROJET DE RECHERCHE
DE THÈSE

Nom de l'étudiant(e) : John Kayser
Code Permanent : KAYJ20036805

Nom de la directrice : Sylvie Cossette
Codirectrice : José Côté

Titre du projet : Évaluation d'une intervention infirmière personnalisée délivrée via le Web favorisant de la marche à pied après un événement cardiaque.

PROBLÉMATIQUE

Les syndromes coronariens aigus sont parmi les plus importantes causes de mortalité cardiaque et parmi les premières raisons de l'utilisation des soins et des services de santé au Canada. Le counseling en matière d'activité physique est une des composantes de base des interventions en prévention secondaire puisque l'augmentation du niveau d'activité physique est associée avec une réduction des facteurs de risque cardiaque et du risque de la mortalité cardiaque, une amélioration de la qualité de vie et une réduction de l'utilisation des soins de santé. Les interventions délivrées via le WEB offrent des alternatives novatrices en prévention secondaire qui soient accessibles dans les milieux de vie. Cependant, il y a peu d'études cliniques randomisées qui évaluent, chez les patients vivant avec une maladie coronarienne, des interventions délivrées via le WEB qui offrent des conseils et un soutien personnalisés selon le degré de motivation et confiance du patient à augmenter le temps de la marche à pied suite à un syndrome coronarien aigu. Le but du projet est d'évaluer les effets d'une intervention infirmière personnalisée délivrée via le Web sur le niveau de la marche à pied auprès d'une clientèle qui présente un syndrome coronarien aigu.

Université de Montréal

Faculté des sciences infirmières

STRATÉGIE(S) D'INTERVENTIONS

Un essai clinique randomisé sera réalisé. Les participants (N=144) seront randomisés dans 2 groupes parallèles: 1) les participants du groupe expérimental recevront, pendant 4 semaines, TAVIEenM@RCHE, une intervention délivrée via le WEB consistant des conseils et un soutien personnalisés selon le degré de motivation et confiance du patient et 2) ceux du groupe contrôle auront accès à une liste de sites web publics. Les patients seront recrutés durant leur hospitalisation pour un syndrome coronarien. Seuls les patients qui faisaient moins de 150 minutes d'activités physiques avant leur hospitalisation seront retenus. Les mesures de base seront complétées à 3 semaines après leur congé de l'unité coronarienne. L'intervention débutera après la randomisation 4 semaines suite au congé. Les mesures de résultats seront complétées à 5 et 12 semaines après la randomisation. Le résultat primaire est l'augmentation du nombre de pas par jour mesuré par un accéléromètre entre la mesure de base et 12 semaines. Les résultats secondaires incluent l'augmentation de la dépense énergétique de la marche à pied et de l'activité physique auto rapportée. Des résultats exploratoires incluent des améliorations dans la qualité de vie, la fréquence d'angine, le tabagisme, l'adhésion au traitement pharmacologique et l'entrée en prévention secondaire ainsi que les visites à l'urgence et les hospitalisations. Le degré d'effet des variables de motivation et de confiance sur le nombre de pas par jour sera aussi exploré. Dépendamment de la capacité de TAVIEenM@RCHE à favoriser la marche, cette intervention pourrait constituée un outil complémentaire aux soins usuels et bénéfiques pour la santé des personnes vivant avec une maladie coronarienne au Québec et le Canada.

Signature de l'étudiante — Date: le 2 juillet 2015

Ce projet de recherche est conforme aux règles d'éthique concernant l'utilisation des êtres humains en expérimentation.

John Kayser	le 2 juillet 2015	Sylvie Cossette
Étudiante	Date	Directeur de recherche

Cette thèse sera rédigée par articles : Oui ☑ Non ☐

Université de Montréal

Faculté des sciences infirmières

SIGNATURES

Suite à l'évaluation de ce projet de recherche effectué dans le cadre d'une thèse de doctorat, nous considérons qu'il répond aux exigences d'un travail scientifique.

NOM : Anne Bourbonnais Signature : A Bourbonnais
Présidente du comité d'approbation

NOM : Margaret Purden Signature : Margaret Purden
Membre du comité d'approbation

Date : 2 juillet 2015
